# Metformin inhibits hepatocellular glucose, lipid and cholesterol biosynthetic pathways by transcriptionally suppressing steroid receptor coactivator 2 (SRC-2)

**DOI:** 10.1038/srep16430

**Published:** 2015-11-09

**Authors:** Andre Madsen, Olivera Bozickovic, Jan-Inge Bjune, Gunnar Mellgren, Jørn V. Sagen

**Affiliations:** 1 Hormone Laboratory, Haukeland University Hospital, N-5021 Norway; 2 Department of Clinical Science, University of Bergen, N-5020 Norway; 3 KG Jebsen Center for Diabetes Research, University of Bergen, N-5020 Norway

## Abstract

The ability of the anti-diabetic drug metformin to inhibit anabolic processes including gluconeogenesis and lipogenesis is partly attributable to activation of the AMP-activated protein kinase (AMPK) pathway. The p160 steroid receptor coactivator 2 (SRC-2) is a key regulator of cellular metabolism and drives expression of the gluconeogenic enzyme glucose-6-phosphatase (G6Pc). Here, we uncovered a role for SRC-2 in the metabolic reprogramming imposed by metformin. In FaO cells, metformin dose-dependently reduced mRNA expression of SRC-2. Microarray analysis of metformin-treated cells revealed an overrepresentation of downregulated genes involved in biosynthesis of lipids and cholesterol. Several metformin-regulated genes including fatty acid synthase (FASN) were validated as transcriptional targets of SRC-2 with promoters characterized by sterol regulatory element (SRE) binding protein (SREBP) recognition sequences. Transactivation assays of the FASN promoter confirmed that SRC-2 is a coactivator of SREBP-1. By suppressing SRC-2 at the transcriptional level, metformin impeded recruitment of SRC-2 and RNA polymerase II to the G6Pc promoter and to SREs of mutual SRC-2/SREBP-1 target gene promoters. Hepatocellular fat accretion was reduced by metformin or knock-down of both SRC-2 and SREBP-1. Accordingly we propose that metformin inhibits glucose and lipid biosynthesis partly by downregulating SRC-2 gene expression.

The p160 steroid receptor coactivator (SRC) family consists of the three distinct members SRC-1/NCOA1, SRC-2/NCOA2/GRIP1/TIF2 and SRC-3/NCOA3/AIB1 that aid in the function of nuclear hormone receptors and transcriptional regulation of target genes. The SRCs thus regulate metabolism and a variety of cellular processes by facilitating transcription of hormonally regulated target genes^1^,^2^,^3^. SRC-2 regulates physiology and metabolism in a tissue-specific manner and is accordingly subject to regulation by several cell signaling pathways and even circadian events^4^,^5^,^6^,^7^. Previous studies have demonstrated that SRC-2 contributes to obesity and insulin resistance^8^, fat accretion^4^,^7^, hepatic gluconeogenesis^9^,^10^ and biosynthesis of lipids^4^,^9^,^10^ and cholesterol^6^,^9^,^10^ at the transcriptional level. It has previously been shown that SRC-2 liver knock-out mice exhibit fasting hypoglycemia due to reduced hepatic expression of the rate-limiting gluconeogenic enzyme glucose-6-phosphatase (*G6pc*)^10^. This observation was coupled with the finding that SRC-2 acts as a coactivator of the orphan nuclear receptor RAR-related orphan receptor alpha (RORα) on the proximal G6pc promoter^10^. Microarray profiling of liver from SRC-2 knock-out mice revealed reduced expression of *Srebp1* and several key metabolic enzymes involved in gluconeogenesis (*G6pc*), lipogenesis (*Fasn*, *Elovl6*) and cholesterol synthesis (*Hmgcr*, *Hmgcs1*, *Nsdhl*, *Cyp51*)^11^. Notably, the sterol regulatory element binding protein 1 (SREBP-1) represents a master regulator of biosynthesis of lipids and cholesterol^12^. It was recently demonstrated that SRC-2 is activated in response to phosphorylation by the nutrient-sensor mammalian target of rapamycin complex 1 (mTORC1) and this event enables SRC-2 coactivation of SREBP-1 to promote lipogenesis and survival of particularly lipid-reliant prostate cancer cell models^4^.

Metformin, a synthetic biguanide drug, remains the preferred pharmacologic treatment for type 2 diabetes^13^,^14^. The ability of metformin to suppress hepatic gluconeogenesis and other anabolic pathways including lipid and cholesterol biosynthesis is partly attributed to transient inhibition of the mitochondrial respiratory chain complex I and indirect activation of the energy-sensing AMP-activated protein kinase (AMPK) pathway^15^,^16^,^17^. Administration of metformin is associated with activation of AMPK and transcriptional repression of hepatic gluconeogenic enzymes^18^. Furthermore, AMPK inhibits lipogenesis by reducing activity of acetyl-CoA carboxylase^14^ and downregulating SREBP-1 and its target genes including fatty acid synthase (FASN)^19^. Several studies also support a role for metformin in the treatment of non-alcoholic fatty liver disease (NAFLD)^20^,^21^,^22^,^23^. The pleiotropic AMPK pathway regulates cellular energy balance by modulating metabolism and this is partly achieved by inhibition of mTORC1 activity^16^,^24^. Interestingly, metformin-induced inhibition of both mTORC1 activity and gluconeogenic gene expression is retained in the absence of the catalytic AMPK subunits^25^,^26^. Thus, unlike the AMP analogue 5-aminoimidazole-4-carboxamide ribonucleotide (AICAR), the precise mechanisms by which metformin exerts its effects on hepatocyte metabolism remain elusive and several reports suggest that the central therapeutic properties of metformin in fact are mediated independently of the AMPK pathway^25^,^27^,^28^,^29^.

Liver knock-out SRC-2 mice are characterized by reduced expression of rate-limiting enzymes pertaining to gluconeogenesis and biosynthesis of lipids and cholesterol^4^,^6^,^9^. This study aimed to determine whether the seemingly overlapping metabolic alterations induced by metformin may involve a downregulation of SRC-2.

## Results

### Metformin inhibits expression of SRC-2

**Figure 1:**
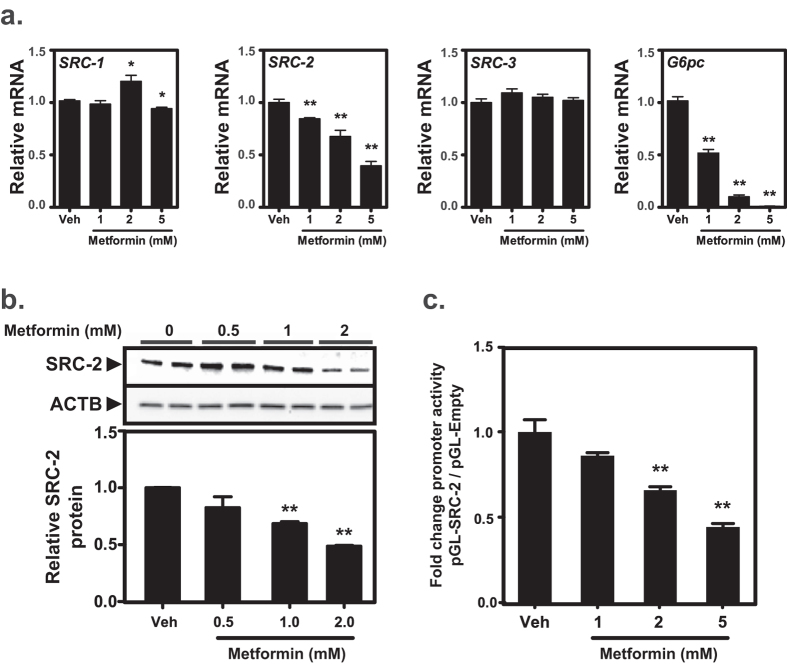
Metformin inhibits expression of SRC-2. (**a**) The effect of vehicle (Veh, water) or metformin treatment on the endogenous mRNA expression levels of steroid receptor coactivators SRC-1, SRC-2 and SRC-3 in FaO hepatoma cells was determined by RT-qPCR. Metformin is known to downregulate *G6pc* expression and this gene was included as a positive control. Target gene expression was normalized to reference gene *Rpl4* and represented as mean fold change relative to vehicle treatment ± SEM of biological triplicates. (**b**) FaO cells were treated as indicated and biological duplicates of 30 μg lysate were analyzed by Western blotting using antibodies against SRC-2 and ACTB load control. Volumetric densitometry band analysis of SRC-2 protein was normalized to ACTB. Data present mean relative SRC-2 protein amount ± SEM of biological duplicates in three independent experiments. **(c)** HepG2 cells were transfected with luciferase reporter constructs under transcriptional control of the bases −1500/+1 of the human SRC-2 promoter (pGL-SRC-2) or no promoter (pGL-empty). Reporter transactivation was measured by luminescence and is presented as the mean ratio between the two constructs, relative to vehicle treatment ± SEM of biological triplicates. *p < 0.05; **p < 0.01.

The established effects of metformin on hepatocellular metabolism led us to investigate whether metformin affects the expression of members of the steroid receptor coactivator family. The effects of increasing doses of metformin on relative expression levels of the steroid receptor coactivators SRC-1, SRC-2 and SRC-3 were assessed in the rat FaO hepatoma cell line (Fig. 1a). Whereas mRNA levels of SRC-1 and SRC-3 remained essentially unchanged in response to treatment with metformin, we observed a dose-dependent downregulation of SRC-2 mRNA. This range of metformin concentration (0–5 mM) was not cytotoxic to the cells and did not significantly affect mRNA levels of the house-keeping gene *Rpl4*. Expression levels of the gluconeogenic enzyme *G6pc* was included as positive control of the pharmacological effect of metformin. Since metformin significantly reduced SRC-2 mRNA we next investigated whether this downregulation also would affect endogenous SRC-2 protein level (Fig. 1b). As confirmed by densitometry of Western blots from three independent experiments, metformin concentrations at or above 1 mM caused a significant reduction of SRC-2 protein relative to beta-Actin (ACTB) in FaO cells. The above findings were replicated in HepG2 cells (data not shown). HepG2 cells were used for transactivation assays due to more effective plasmid transfection. Transactivation of a luciferase reporter construct under transcriptional control of the endogenous SRC-2 promoter (containing bases −1500 to +1 relative to the SRC-2 transcription start site) was used to detect whether metformin downregulates SRC-2 at the transcriptional level. This reporter was dose-dependently inhibited by metformin relative to the control pGL-basic reporter (Fig. 1c). Taken together, these results demonstrate that treatment of cells with metformin decreases SRC-2 mRNA and protein levels.

### Metformin inhibits transcription of SRC-2 target genes involved in biosynthesis of lipids and cholesterol

**Figure 2:**
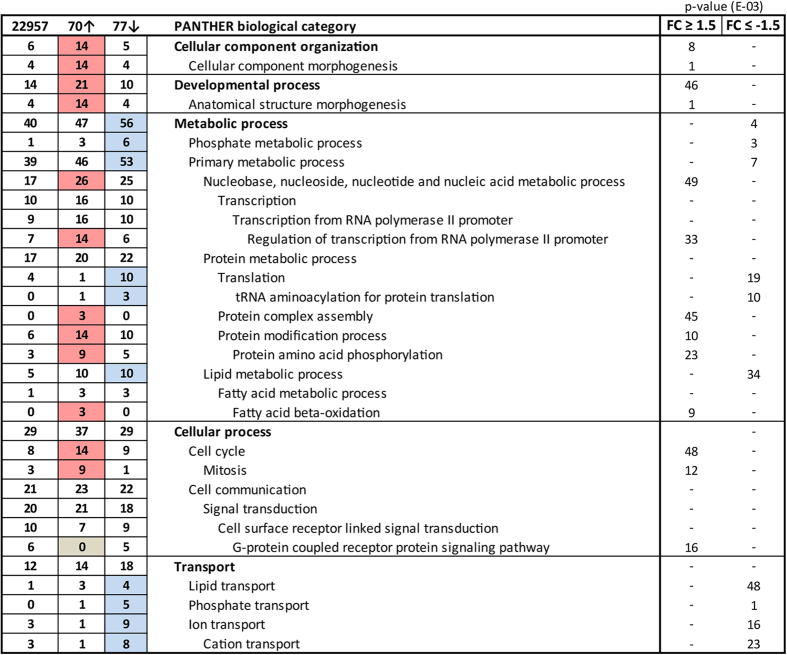
Metformin-induced changes in the transcriptome. Based on the significance analysis of microarrays, genes that were differentially regulated by metformin were ordered into PANTHER biological categories. Only genes exhibiting a fold change (FC) equal to or greater than 1.5 (FC ≥ 1.5; upregulated) or equal to or less than −1.5 (FC ≤ −1.5; downregulated) and FDR ≤ 0.2 were included. Red and blue cells annotate significant overrepresentation of upregulated and downregulated gene categories, respectively.

**Figure 3:**
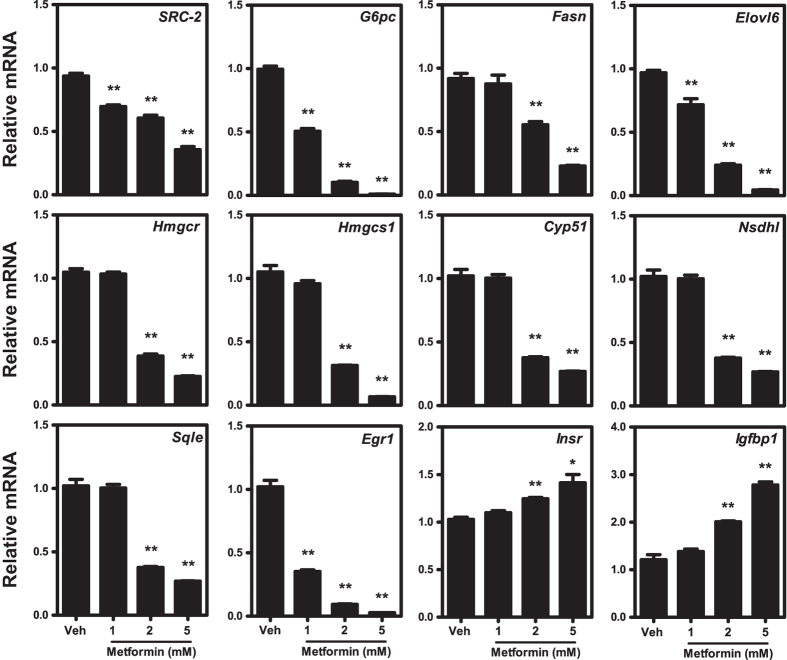
Downregulation of SRC-2 and its target genes by metformin. FaO cells were treated for 24 hours with increasing concentrations of metformin prior to RT-qPCR analysis of endogenous mRNA expression levels of the indicated genes. Expression levels of *Insr* and *Igfbp1* were included as positive controls of metformin-upregulated genes. Gene expression was normalized to the reference gene *Rpl4* and presented as mean fold change relative to vehicle treatment ± SEM of biological triplicates. One representative out of three independent experiments is shown. Where indicated, **p < 0.01.

**Figure 4:**
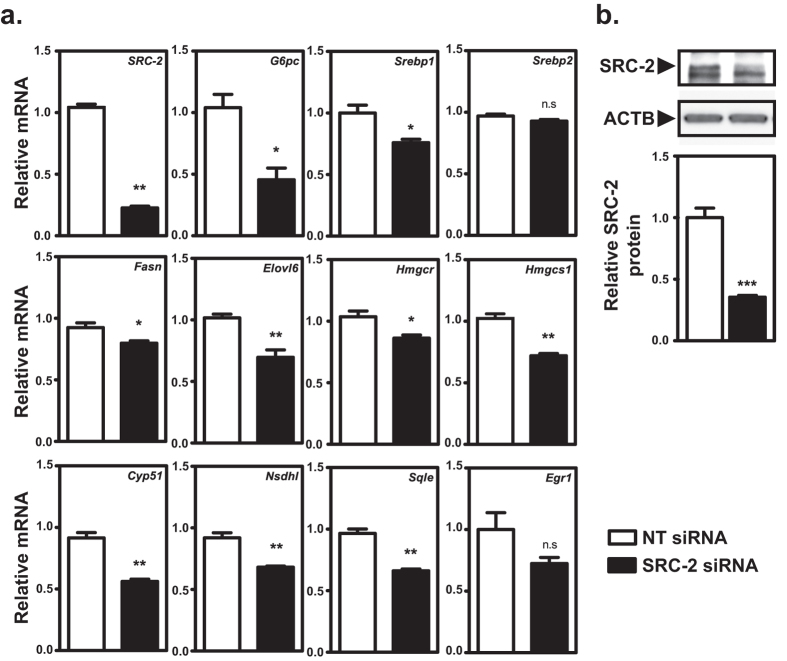
Knock-down of SRC-2 reduces target gene expression. (**a**) Quantification of endogenous mRNA levels of SRC-2 and its target genes in FaO cells were measured by RT-qPCR, 72 hours after transfection with non-targeting (white bars) or SRC-2 (black bars) siRNA. Gene expression was normalized to reference gene *Rpl4* and presented as mean fold change relative to transfection with NT siRNA ± SEM of biological triplicates of a representative experiment. **(b)** Protein levels of SRC-2 and load control ACTB were assessed by Western blot 72 hours after transfection of FaO cells with SRC-2 or NT siRNA. Quantification of protein bands was determined by densitometry. Data is presented as the relative SRC-2 protein amount ± SEM of nine biological replicates, representative of two independent experiments. *p < 0.05; **p < 0.01, ***p<0.001; n.s not significant.

In order to elucidate how metformin affects the hepatocellular transcriptome we performed a significance analysis of microarray based on FaO cells treated with vehicle or 5 mM metformin and categorized the genes into respective biological processes using the PANTHER database (Fig. 2). As expected, mainly genes related to metabolic processes were differentially regulated by metformin and among the upregulated genes there was a significant overrepresentation of factors involved in fatty acid β-oxidation (p < 0.01). As for genes that were subject to transcriptional downregulation by metformin, there was a significant statistical overrepresentation of factors involved in lipid metabolic processes (p < 0.05). Notably, several of the genes included in this category have previously been identified as transcriptional target genes of SRC-2 (Table 1). We next performed a separate qPCR validation of the microarray and overlapping SRC-2 target genes in which FaO hepatoma cells were incubated with increasing concentrations of metformin. Combining the annotated genes from Table 1 with other known SRC-2 target genes^11^, we observed that metformin dose-dependently downregulated mRNA levels of SRC-2 target genes involved in gluconeogenesis (*G6pc*), biosynthesis of lipids (*Fasn, Elovl6)* and cholesterol (*Hmgcr, Hmgcs1, Sqle, Nsdhl*, *Cyp51* and *Egr1*) (Fig. 3). In line withour microarray data, we found that the insulin receptor (*Insr*) was upregulated by metformin, and *Igfbp*, a known metformin-inducible gene^30^,^31^, was included as a positive control. In order to verify that these genes indeed represent SRC-2 target genes we performed transient siRNA knock-down of SRC-2 in FaO cells. Endogenous gene expression levels of all genes were measured by RT-qPCR 72 hours after siRNA transfection (Fig. 4a). We observed that knock-down of SRC-2 reduced *G6pc* expression and also significantly lowered mRNA levels of the included genes relating to lipid and cholesterol biosynthesis, with the only exception of *Egr1*. In the microarray, the *Srebp1* gene was omitted due to the unsatisfactory FDR associated with this gene. Notably, *Srebp1* but not *Srebp2* has been previously identified as a SRC-2 target gene^11^ and we were only able to detect a moderate decrease of *Srebp1* mRNA in response to knock-down of SRC-2. To validate that the observed changes in gene expression were indeed caused by the absence of SRC-2 protein we performed Western blot experiments to verify that the knock-down procedure had markedly reduced the SRC-2 protein level (Fig. 4b). Taken together, these results indicate that metformin causes transcriptional repression of key metabolic genes, of which several represent SRC-2 target genes.

### Metformin inhibits recruitment of SRC-2 to target gene promoters

**Figure 5:**
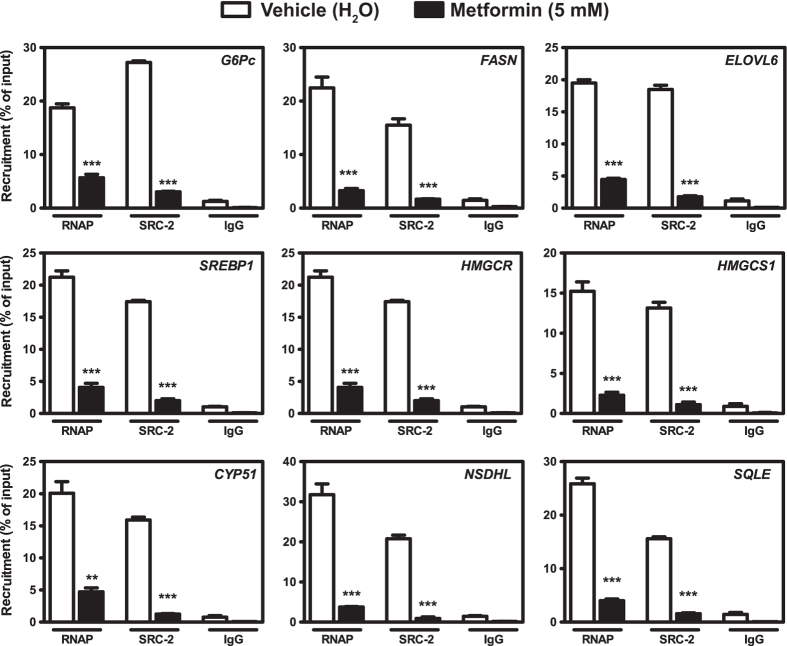
Recruitment of SRC-2 and RNA polymerase to target gene promoters. Chromatin immunoprecipitation of RNA polymerase II (RNAP) and SRC-2 in HepG2 cells treated with vehicle (white bars) or 5 mM metformin (black bars). IgG represents assay background noise. Eluted sample DNA was subjected to qPCR amplification of regions overlapping known SREBP-1 binding sites at the proximal promoters of indicated target genes. For G6Pc, primers were designed to amplify the genomic promoter region flanking the proximal RORE. Signal quantification was normalized to that of respective treatment 1% input samples and data is presented as mean recruitment (% of input) ± SEM of biological triplicates in one representative out of three independent experiments. *p < 0.05; **p < 0.01; ***p < 0.001.

Gene expression is preceded by binding of transcriptional activators and associated coactivators that ultimately recruit RNA polymerase II (RNAP) to target gene promoters. Bioinformatic analyses using Genomatix, UCSC and ENCODE databases revealed that sterol regulatory elements (SREs) were a common transcription factor binding motif at the proximal promoters of the above SRC-2 target genes, including *Srebp1* itself, but with the exception of *G6pc*. On the proximal *G6pc* promoter, however, it has previously been shown that SRC-2 is a coactivator of the nuclear receptor RORα^10^. In order to determine whether metformin reduces recruitment of SRC-2 to target gene promoters we performed chromatin immunoprecipitation (ChIP) with respect to SRC-2 and RNAP protein and designed qPCR primers to amplify regions of proximal SREs of respective target gene promoters. For the *G6pc* promoter, primers were designed to amplify the relevant proximal RORα response element (RORE). Due to the inability of any tested ChIP-grade antibody to immunoprecipitate the rat SRC-2 antigen, HepG2 cells were used for this assay. HepG2 cells were incubated with vehicle (water) or metformin (5 mM) for 24 hours and ChIP end-point qPCR demonstrated that recruitment of both RNAP and SRC-2 to target gene promoters (*G6PC, FASN, ELOVL6, HMGCR, HMGCS1, CYP51, NSDHL, SQLE* and *SREBP1*) was significantly reduced upon treatment with metformin (Fig. 5). These results are in agreement with the above findings, that both metformin and transient knock-down of SRC-2 reduce expression of the corresponding genes. Notably, the levels of unspecific recruitment observed with negative control IgG antibody appeared at very late qPCR cycles for all promoters. Thus, the insignificantly small background noise did not influence interpretation of results in terms of absolute quantification or by normalization according to the [Ct (Sample)] – [Ct (IgG)] subtraction method.

### SRC-2 coactivates SREBP-1 at the FASN promoter

**Figure 6:**
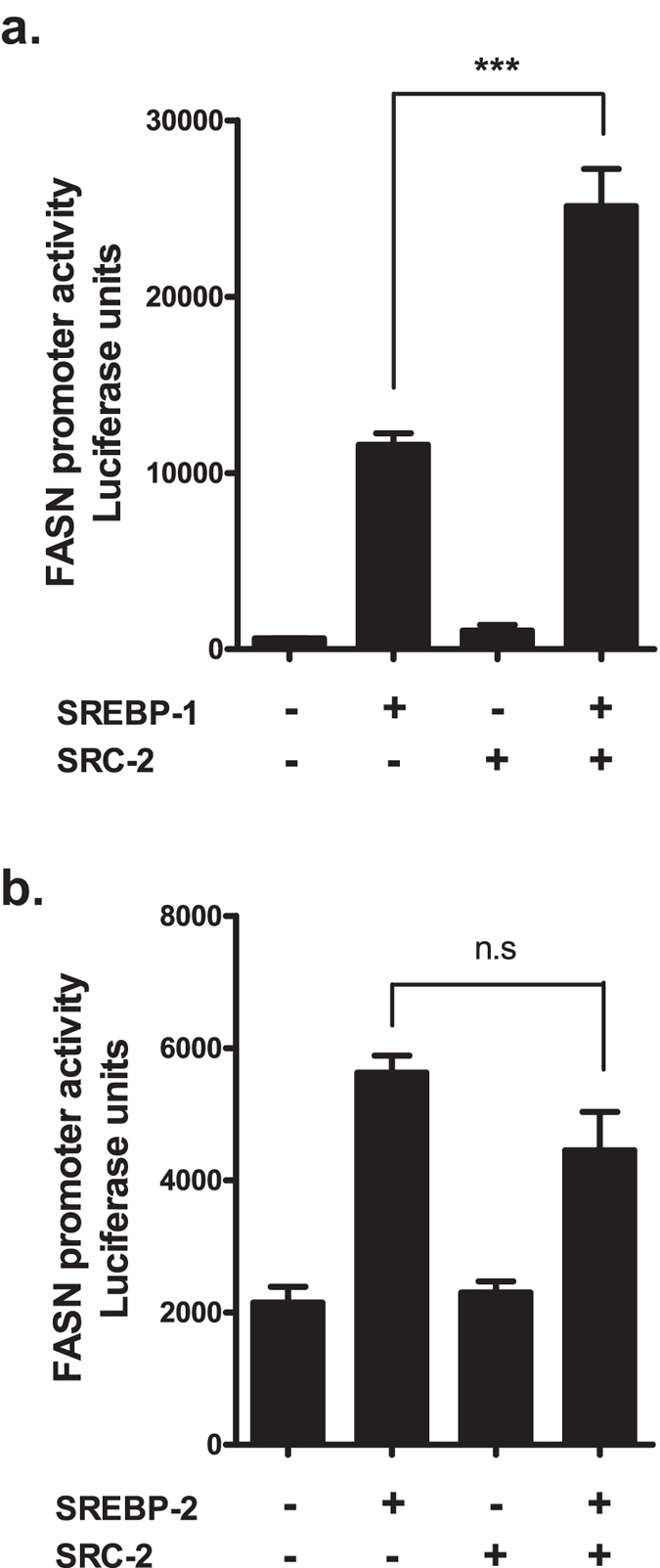
SRC-2 is a coactivator of SREBP-1. Transactivation of a luciferase reporter construct under transcriptional control of the endogenous −220/+25 FASN promoter in response to combinatorial overexpression of SRC-2 and (**a**) SREBP-1 or (**b**) SREBP-2. Data are presented as mean arbitrary luciferase units ± SEM of biological quadruplicates. The presence or absence of coactivation synergy is illustrated. ***p ≤ 0.001; n.s not significant.

In light of the recent discovery that SRC-2 acts as a coactivator of SREBP-1 in prostate cancer cells^32^ we wanted to examine whether this also occurred in a hepatocellular system. Additionally, we investigated whether SRC-2 could coactivate the related SREBP-2. A luciferase reporter construct under transcriptional control of the proximal FASN promoter, containing bases −220/+25 relative to transcription start site, was overexpressed in conjunction with full-length SREBPs and SRC-2 in HepG2 cells. The combined overexpression of SREBP-1 and SRC-2 synergistically stimulated reporter transactivation, suggesting that SRC-2 indeed coactivates SREBP-1 at the FASN promoter (Fig. 6a). In contrast, this effect was not observed with overexpression of SRC-2 and SREBP-2, suggesting that SRC-2 does not act as a coactivator for SREBP-2 in this context (Fig. 6b). It is plausible that SRC-2 coactivation of SREBP-1 is also relevant with respect to other SREBP-1 target gene promoters.

### Knock-down of SRC-2 and Srebp1, and treatment with metformin reduces hepatocellular fat accumulation

**Figure 7:**
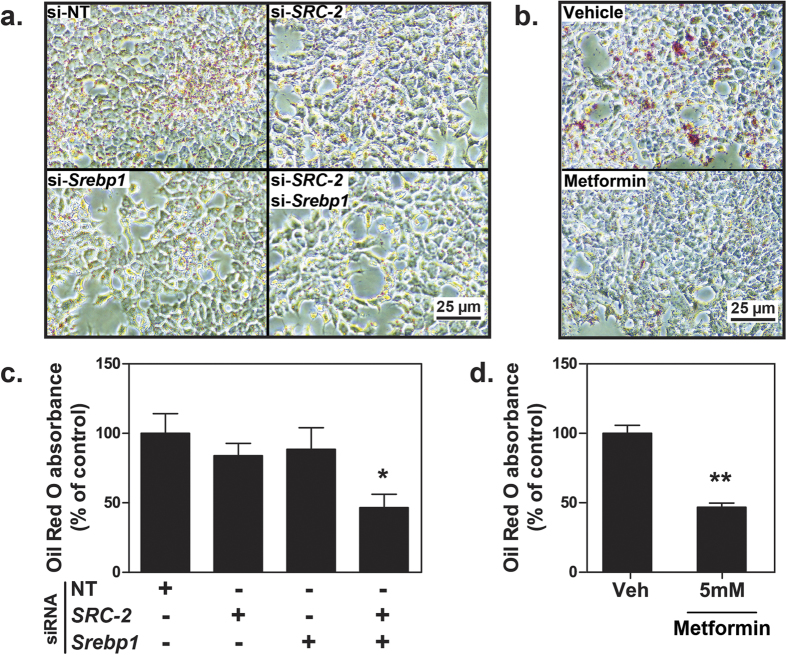
Inhibition of hepatocellular fat accretion. (**a**) Oil Red O staining of FaO hepatoma cells at 40x magnification after combinatorial knock-down with non-targeting (NT), SRC-2 and *Srebp1* siRNA. Cells were starved in serum-free medium for 48 hours prior to introduction of medium supplemented with 10% FBS and 2 mM glutamine for 24 additional hours. Scale bar: 25 μm. (**b**) Oil Red O staining of FaO cells at 40x magnification following starvation for 48 hours prior to introduction of medium supplemented with 10% FBS, 2 mM glutamine and vehicle (Veh, water) or increasing doses of metformin for 24 additional hours. Scale bar: 25 μm. (**c**) Quantitative analysis of Oil Red O content of cells from experiment (**a**) was done by measuring optical density of eluate at 490 nm. By convention, total Oil Red O content from cells transfected with NT siRNA was defined as 100% and all other samples were normalized accordingly. (**d**) Quantitative analysis of Oil Red O content of cells from the experiment (**b**). Data are presented as percent mean absorbance relative to cells treated with vehicle ± SEM of biological triplicates from one representative out of three or more independent experiments. *p < 0.05; **p < 0.01.

In order to clarify the physiologic relevance of our above results, we next investigated whether the absence of SREBP-1 and its coactivator SRC-2 would affect actual lipogenesis and cellular lipid content. Therefore we knocked down *Srebp1* and SRC-2 in FaO hepatoma cells and starved the cells in serum-free medium for 48 hours prior to re-introduction of supplemented medium for 24 additional hours to facilitate lipogenic fat accretion. End-point Oil Red O staining demonstrated that cellular lipid content was reduced when *Srebp1* and SRC-2 were knocked down simultaneously (Fig. 7a). Optical density quantitative measurements of the total cellular Oil Red O content confirmed that lipid levels were significantly reduced when both *Srebp1* and SRC-2 were knocked down (Fig. 7b). We also starved FaO cells in serum-free medium for 48 hours prior to reintroduction of supplemented medium containing vehicle (water) or 5 mM metformin for 24 additional hours. Subsequent Oil Red O staining (Fig. 7c) and optical density quantification (Fig. 7d) demonstrated that cellular lipid contents were significantly reduced upon treatment with metformin compared to the vehicle control. These results demonstrate that Srebp1 and SRC-2 contribute to stimulate hepatocellular lipogenesis, whereas metformin has an inhibitory effect on this same process.

## Discussion

Previous studies have demonstrated that hepatic SRC-2 transcriptionally promotes energy-demanding pathways including gluconeogenesis and biosynthesis of fatty acids and cholesterol. Here, we demonstrate that the pharmacological effects of metformin extend to inhibit expression of SRC-2 and, in turn, SRC-2 target genes including rate-limiting enzymes pertaining to gluconeogenesis (*G6pc*) and biosynthesis of lipids (*Fasn*) and cholesterol (*Hmgcr* and *Hmgcs1*). Previously it was shown that SRC-2 can act as a coactivator SREBP-1 to promote lipogenesis and survival of prostate cancer cells^4^. Metformin and the AMPK pathway are known to inhibit activity of both mTORC1 and SREBP-1^16^,^19^. However, several pharmacological properties of metformin appear to be mediated independently of the AMPK pathway. Interestingly, the ability of metformin to inhibit both gluconeogenic gene expression and mTORC1 activity is retained in absence of the AMPK enzyme^25^,^26^.

Our initial findings show that metformin, in a concentration-dependent manner, causes transcriptional downregulation and thus reduction of the available protein level of SRC-2, and this effect was not observed with SRC-1 or SRC-3. Microarray analysis of FaO hepatoma cells treated with or without metformin revealed significant upregulation of genes involved in fatty acid β-oxidation. Conversely, there was a significant overrepresentation of downregulated genes associated with lipid biosynthesis, of which several were identified as SRC-2 target genes. Combining the latter entry with other metabolically relevant and previously established SRC-2 hepatic target genes identified by Jeong *et al.*^11^, we show that metformin in a dose-dependent manner inhibits transcription of this entire panel of SRC-2 target genes. The observed difference in dose responses for these genes is likely due to metformin differentially modulating the intrinsic activity of a large array of other transcription factors and coregulators on the various promoters. In line with the microarray results and the insulin-sensitizing property of metformin, we also show that metformin stimulates expression of the insulin receptor (*Insr*). Furthermore, the stimulating effect of metformin on expression of the insulin-like growth factor binding protein 1 (*Igfbp1*) has been described previously^30^,^31^. We also demonstrate that transient siRNA knock-down of SRC-2 is sufficient to significantly reduce mRNA expression of indicated SRC-2 target genes, with the exception of Early growth response 1 (*Egr1*). Notably, *Egr1* has been reported to be a transcriptional target of SRC-2^11^,^33^ and is implicated in promotion of obesity and insulin resistance^34^, progression of prostate cancer^35^ and transcriptional regulation of lipid and cholesterol biosynthetic genes^36^. Interestingly, *Egr1* was markedly downregulated when cells were treated with metformin, and this could potentially be related with the recent discovery that metformin administration after prostate cancer diagnosis is associated with decreased mortality^37^.

It has previously been shown that SRC-2 coactivates SREBP-1 and we therefore wanted to investigate whether the reduced SRC-2 expression and protein level in response to metformin treatment also would affect recruitment of SRC-2 to mutual target gene promoters. Sequential recruitment of the general transcription machinery and ultimately RNA polymerase II is a core property of steroid receptor coactivators, including SRC-2^6^. Using ChIP analysis we demonstrate that metformin significantly reduces recruitment of both endogenous SRC-2 and RNA polymerase II to sterol regulatory elements (SREs) located proximally at promoters of the entire panel of SRC-2 target genes. Notably, there is no SRE at the human *G6Pc* promoter. However, SRC-2 is a known coactivator of the nuclear receptor RORα on the *G6Pc* promoter^10^ and we observed that recruitment of SRC-2 to the relevant proximal RORα response element (RORE) was significantly reduced when cells were treated with metformin. It is well established that transcriptional control of coactivators is essential to the regulation of hepatic gluconeogenesis^38^. For example, the inhibitory effect of metformin and AMPK on the CREB regulated transcription factor 2 (CRTC2/TORC2) impedes expression of the master gluconeogenic coactivator PGC-1α^39^. Our observation that metformin inhibits SRC-2 expression is thus reconcilable with the established effect of metformin.

Previous studies have shown that transcription of SREBP-1 is positively regulated by insulin, and that metformin inhibits expression of SREBP-1 *in vivo*^40^. By downregulating both SREBP-1 expression and activity, metformin effectively inhibits lipogenesis and accumulation of hepatic lipids^20^,^40^,^41^. Here, we show in a hepatocellular system that SRC-2 is able to coactivate SREBP-1, but not SREBP-2, and that also SRC-2 is subject to transcriptional suppression by metformin. Our findings complement the established inhibitory effect of metformin on SREBP-1 and provide a new layer of complexity as to how metformin suppresses the transcriptional activity of SREBP-1 and lipogenesis. In particular, a key feature of SREBP-1 is to mediate expression of FASN which is the rate-limiting enzyme in *de novo* lipogenesis. Whereas knock-down of *Srebp1* alone in our system was insufficient to functionally lower hepatocellular fat accretion, we observed a marked decline in intracellular fat content upon simultaneous knock-down of *Srebp1* and SRC-2. This implies that SRC-2 markedly contributes to hepatocellular lipogenesis by coactivating SREBP-1. Metformin alone significantly reduced hepatocellular lipogenic fat accumulation, and this may partly be due to transcriptional suppression of SRC-2.

In conclusion, we provide evidence that treatment of cells with metformin is accompanied by reduced expression of SRC-2, which in turn impedes transcription of gluconeogenic G6Pc and coactivation of SREBP-1 at key metabolic target gene promoters. We propose that the ability of metformin to transcriptionally suppress gluconeogenesis and biosynthesis of lipids and cholesterol is in part due to transcriptional inhibition of SRC-2.

## Methods

### Cell culture, transfection and siRNA

Human HepG2 and rat FaO hepatoma cells were purchased from ATCC and cultured in a 5% CO_2_ incubator at 37 °C. The culture media EMEM (Lonza, Basel, Switzerland) and F-12 HAM (Gibco, Waltham, MA, USA) were supplemented with 10% fetal bovine serum (FBS) (Gibco), 1% penicillin-streptomycin (Sigma, St. Louis, MO, USA) and 2 mM L-glutamine (Lonza). Cells were seeded 24 hours prior to transfection to a standardized density of 50.000 cells/24-well. For indicated experiments, cells were treated for 24 hours with metformin hydrochloride (Sigma) dissolved in sterile-filtered water. Transient plasmid transfections were performed in HepG2 cells using TransIT-LT1 reagent (Mirus, Madison, WI, USA) according to the manufacturer’s protocol. All plasmids were verified by sequencing. The following reporter constructs were used: pGL-basic; pGL-SRC-2 (containing promoter bases −1500/+1) and pFASN-luc (containing promoter bases −220/+25). The following expression vectors were used: pSG5-HA-SRC-2; pSG5-empty; pcDNA3.1-2xFLAG-SREBP-1a and pcDNA3.1-2xFLAG-SREBP-2. For knock-down experiments we employed the TransIT-TKO transfection reagent (Mirus) with rat SRC-2 *(Nuclear receptor coactivator 2*) and rat non-targeting (NT) SMARTpool ON-TARGETplus siRNA (Dharmacon, Lafayette, CO, USA) at 100 nM final concentration. Cells were transfected with siRNA 72 hours prior to further analyses.

### Transactivation assays

Cells were lysed with buffer containing 25 mM Tris Acetate-EDTA (pH 7.8), 2 mM dithiotheitol, 1 mM EDTA, 10% glycerol and 1% Triton X-100 and sample lysate luciferase activity was analyzed using a luciferase kit (BioThema, Handen, Sweden) and a FLUOStar Optima (BMG Labtech, Ortenberg, Germany) luminescence plate reader.

### RT-qPCR analysis of gene expression

Cells were lysed in RLT buffer (Qiagen, Hilden, Germarny) and processed for RNEasy (Qiagen) RNA isolation according to manufacturer’s protocol. Yield concentrations were measured by NanoDrop and 500 ng sample RNA was used for cDNA synthesis using High-Capacity cDNA Reverse Transcription kit (Applied Biosystems, Waltham, MA, USA) according to the manufacturer’s instructions. Synthesized cDNA was diluted 1:10 in water prior to SYBR I Green (Roche, Basel, Switzerland) real-time RT-qPCR of target genes relative to reference gene *Rpl4*. Primer sequences are presented in the Supplementary Table S1. Relative target/*Rpl4* mRNA quantification was calculated using the delta-delta Ct method.

### Microarray

FaO cells were treated with metformin (5 mM) or vehicle (water) for 24 hours prior to RNEasy (Qiagen) RNA purification. Integrity of total RNA (RIN value ≥ 9.0) from n = 3 biological replicates per treatment was verified by Agilent Bioanalyzer prior to sample randomization and microarray (Agilent, Santa Clara, CA, USA) at the Genomics Core Facility, University of Bergen. No sample batch effects were observed. Significance analysis of microarrays was performed with J-Express software (http://jexpress.bioinfo.no/site). Only genes exhibiting a fold-change FC ≤ −1.5 or FC ≥ 1.5 relative to vehicle and false discovery rate (FDR) ≤ 0.2 were included in subsequent analyses. Functional annotations of valid genes and statistical overrepresentation of biological categories were retrieved in accordance with instructions^42^ using the PANTHER online database (pantherdb.org). The PANTHER library of 22957 annotated genes (*Rattus norvegicus*) was used as reference list for calculating statistical overrepresentation. Microarray data is included as Supplementary Dataset.

### Western blotting

Sample cell lysate (30 μg) was loaded onto precast 4–20% Mini-Protean TGX gels (Bio-Rad, Hercules, CA, USA) and subjected to SDS-PAGE prior to iBlot (Invitrogen, Waltham, MA, USA) transfer to nitrocellulose membrane. The following antibodies were used: beta-Actin/ACTB (Abcam, Cambridge, UK, cat.no ab8227); SRC-2 (BD Biosciences, San Jose, CA, USA, cat. no 610985); HA-HRP (Roche, cat. no 12013819001); HRP goat-anti mouse IgG (BD Biosciences, cat. no 554002); HRP goat-anti rabbit IgG (Thermo Scientific, Waltham, MA, USA, cat. no 31460). Immunoblotted membranes were developed using Femto substrate (Thermo Scientific) and analyzed on a ChemiDoc XRS imager (Bio-Rad) equipped with QuantityOne densitometry software. For densitometry analyses, volumetric protein band intensity was normalized to that of ACTB in the same gel lane.

### Chromatin immunoprecipitation (ChIP)

Seeding of 2.0 × 10^6^ HepG2 cells per 92 mm plate was performed one day prior to treatment with either 5 mM metformin or vehicle (water) for 24 hours. Cells were fixated with 1% (v/v) formaldehyde for 10 minutes and further processed using EZ-Magna ChIP kit (Millipore, Billerica, MA, USA) in accordance with the manufacturer’s protocol. Sonication was optimized to 8 minutes (30 second on/off cycles) using a cold-water bath Bioruptor (Diagenode, Denville, NJ, USA). The following antibodies were used in conjunction with Protein A magnetic beads for over-night immunoprecipitation: ChIP-grade anti-RNA Pol II (Millipore #05-623B); normal IgG (Millipore #PP64B); ChIP grade anti-SRC-2 (Abcam ab9261) in quantities of 5 μl per sample. Primers for end-point SYBR Green qPCR of eluted sample DNA were designed to amplify regions flanking proximal sterol regulatory elements (SREs) at target gene promoters. Primer specificities and SRE motifs were verified *in silico* using the UCSC Genome Browser (http://genome-euro.ucsc.edu) and Genomatix software. Primer sequences are listed in Supplementary Table S2. Quantification of samples by qPCR was normalized to parallel qPCR of 1% sonicated lysate input of respective treatments.

### Oil Red O lipid staining

Cells were treated as indicated prior to fixation in 4% formaldehyde in PBS for 5 minutes followed by 1.5 hours with fresh 4% formaldehyde in PBS. Cells were washed twice with sterile water prior to incubation for 5 minutes with 60% isopropanol and being completely dried. Per well (12-well format) 1 ml of Oil Red O working solution (0.30 g Oil Red O in 100 ml 99% isopropanol, diluted 3:2 with sterile water) was added and left for 20 minutes prior to washing of each well 4 times with sterile water. Cells were depicted by Nikon TS100 light microscope and subsequent elution of cellular Oil Red O was done by gently washing of the wells with 1 ml 100% isopropanol. Optical density of eluted Oil Red O was measured at 490 nm.

### Statistical analyses

Unless indicated otherwise, data values are expressed as Mean ± SEM of three or more biological replicates. All experiments were reproduced at least three times. All numerical data presented was considered to be of normal distribution and statistical differences between mean values were evaluated using two-tailed, independent Student’s *t*-test. Differences of *p* ≤ 0.05 (indicated*), *p* ≤ 0.01 (indicated**) or *p* ≤ 0.001 (indicated***) were considered significant. GraphPad Prism v5.0 (GraphPad Software Inc, La Jolla, CA, USA) was used for statistical calculations and graphical presentation of data.

## Additional Information

**How to cite this article**: Madsen, A. *et al.* Metformin inhibits hepatocellular glucose, lipid and cholesterol biosynthetic pathways by transcriptionally suppressing steroid receptor coactivator 2 (SRC-2). *Sci. Rep.* **5**, 16430; doi: 10.1038/srep16430 (2015).

## Supplementary Material

Supplementary Information

Supplementary Dataset 1

## Figures and Tables

**Table 1:** Transcriptional downregulation of lipid metabolic process genes by metformin.

**Gene**	**Description**	**FC**	**Biosynthetic pathway**
Elovl6*	Elongation of very long chain fatty acids protein 6	−4.9	Lipid biosynthesis
Fasn*	Fatty acid synthase	−2.4	Lipid biosynthesis
Hmgcr*	3-hydroxy-3-methylglutaryl-CoA reductase	−1.7	Cholesterol biosynthesis
Sqle*	Squalene monooxygenase	−2.8	Cholesterol biosynthesis
*Pigm*	Phosphatidylinositol glycan anchor biosynthesis class M	−4.6	Glycosylphosphatidyl-inositol biosynthesis
*Hsd11b2*	Corticosteroid 11-beta-dehydrogenase isozyme 2	−2.7	Cortisol metabolism
*Star*	Steroidogenic acute regulatory protein	−4.6	Steroid hormone biosynthesis
*Gpd1*	Glycerol-3-phosphate dehydrogenase	−2.1	Glycerol metabolism
*Srd5a1*	3-oxo-5-alpha-steroid 4-dehydrogenase 1	−2.4	Steroid hormone biosynthesis
*Fabp2*	Fatty acid-binding protein	−4.5	Fatty acid transport

Gene entry comprising the statistically overrepresented microarray category “lipid metabolic process” (*p* < 0.05). Genes marked with *asterisks indicate previously established SRC-2 target genes^11^. Downregulation of the indicated genes in FaO hepatoma cells after treatment with metformin is annotated by the negative fold change (FC).
