# Supplementary material for: Metformin inhibits hepatocellular glucose, lipid and cholesterol biosynthetic pathways by transcriptionally suppressing steroid receptor coactivator 2 (SRC-2)
Source: Sci Rep. 2015 Nov 9;5:16430. doi: 10.1038/srep16430 (PMC4637908; doi:10.1038/srep16430)
Supplement: Supplementary Information [file srep16430-s1.pdf]

SUPPLEMENTARY INFORMATION

Metformin inhibits hepatocellular glucose, lipid and cholesterol biosynthetic pathways by transcriptionally suppressing steroid receptor coactivator 2 (SRC-2)

Andre Madsen[1,2], Olivera Bozickovic[1,2], Jan-Inge Bjune[1,2], Gunnar Mellgren[1,2,3] and Jørn V. Sagen[1,2,3]

[1]Hormone Laboratory, Haukeland University Hospital, N-5021 Norway

[2]Department of Clinical Science (K2), University of Bergen, N-5020 Norway

[3]KG Jebsen Center for Diabetes Research, University of Bergen, N-5020 Norway

Supplementary Table S1. Listed qPCR primers for amplification of target gene mRNA, *Rattus norvegicus* (FaO cells)

Target mRNA	Direction	Primer sequence
SRC-1/*Ncoa1*	Forward	TGCTCCCGAGGAGGTTAAA
	Reverse	ATCAAACTGGTCAAGGTCAGC
SRC-2/*Ncoa2*	Forward	TGATAGAGCTCTGGGGATACCA
	Reverse	GAACTGCTCCGGATCCAC
SRC-3/*Ncoa3*	Forward	GGGGCTGAGCTGTGAGTTT
	Reverse	CCACTGTTCAGCGAGTCTTTT
G6pc	Forward	CTCACTTTCCCCATCAGGTG
	Reverse	GAAAGTTTCAGCCACAGCAA
Rpl4	Forward	CGATATGCCATCTGTTCTGC
	Reverse	TTCAACCACCAAAGGCAGTT
Srebp1	Forward	CATCGCAAACAAGCTGACC
	Reverse	CCAGATCCAGGTTTGAGGTG
Srebp2	Forward	CCTGGTGTACCTGGGCAAT
	Reverse	GGCCACCTCAGTCCTGTTAT
Fasn	Forward	GGCCACCTCAGTCCTGTTAT
	Reverse	AGGGTCCAGCTAGAGGGTACA
Elovl6	Forward	ATGGATGCAGGAAAACTGGA
	Reverse	GCCCGCTTGTTCATCAGA
Hmgcr	Forward	GACCTTTCTAGAGCGAGTGCAT
	Reverse	GCTATATTCTCCCTTACTTCATCCTG
Hmgcs1	Forward	CAGGCCTACAGGTGGAGTTG

	Reverse	GCATGTGTGTCCCACGAA
Cyp51	Forward	TGAGGCCTCCTATAATGACCA
	Reverse	ATGTCCTGGAGGAATGGTGT
Nsdhl	Forward	TCTCGAGATGCAGGTCTGG
	Reverse	ACGTCCAGAAAGGGATTGG
Sqle	Forward	CTGTCAGAATGCTCGTCTGC
	Reverse	CGCATCTCCCAGAAGTAGGA
Egr1	Forward	ACGAGCACCTGACCACAGA
	Reverse	GGGTAGTTTGGCTGGGATAAC
Insr	Forward	CAGAAAAACCTCTTCAGGCAAT
	Reverse	TTCAAGGGATCTTCGCTTTC
Igfbp1	Forward	AATGGATTTTATCACAGCAAACAG
	Reverse	CATGGGTAGACACACCAGCA

Supplementary Table S2. Listed qPCR primers for amplification of genomic proximal target promoters in relation to chromatin immuoprecipitation experiments, *Homo sapiens* (HepG2 cells)

**Target promoter**	**Direction**	**Primer sequence**
CYP51	Forward	TTGCCCCAGGTCTCCTACTA
	Reverse	ACCACGGCTTCACAGAGTGT
ELOVL6	Forward	CGCCAAGGGTTAATTTCTCA

	Reverse	CCATCACCCTTTTTACTCGTCT
FASN	Forward	AGAGGCAGGGTCCCAAAG
	Reverse	GTGAGCATGGGTGCTGAGTA
G6PC	Forward	CGTGGTTTTTGAGTCCAAAGAT
	Reverse	CCCCTGTTTTATATGCCCTGT
HMGCR	Forward	TCAAGGTCGGGAGTGATGAT
	Reverse	CCCATTTTAACTGCTGAGATCC
HMGCS1	Forward	TGCTAGGCAACCTGACAGAC
	Reverse	CGCTGGAGAGATGGTCAAAT
NSDHL	Forward	CTGGGTTGACCTAAGGCTGA
	Reverse	CCAACACGCAAGGTAATCGT
SQLE	Forward	AGGGATGCTGGTGAGGAAG
	Reverse	CTTCCCCACCGATAAAAGGT
SREBP1	Forward	AAGCATCATTAGGCCCATGT
	Reverse	TAACTGCTGGGCCAGTTACA
